# “Gender-based Violence (GBV) and HIV, they are like sister and brother”: barriers and facilitators to GBV screening and referral in public HIV treatment settings in Uganda

**DOI:** 10.1186/s12913-023-10400-2

**Published:** 2023-12-11

**Authors:** Dorothy Thomas, Alisaati Nalumansi, Mira Reichman, Mine Metitiri, Brenda Kamusiime, Vicent Kasiita, Grace K Nalukwago, Joseph Kibuuka, Lylianne Nakabugo, Florence Nambi, Timothy Muwonge, Jane Simoni, Elizabeth T. Montgomery, Norma Ware, Monique A Wyatt, Andrew Mujugira, Renee Heffron

**Affiliations:** 1 Department of Global Health, University of Washington, Seattle, WA USA; 2 Infectious Diseases Institute, Makerere University, Kampala, Uganda; 3 Department of Psychology, University of Washington, Seattle, WA USA; 4 Women’s Global Health Imperative, RTI International, Berkeley, CA USA; 5 Harvard Medical School, Boston, MA USA; 6 Brigham and Women’s Hospital, Boston, MA USA; 7 Harvard Global, Cambridge, MA USA; 8 Department of Medicine, University of Alabama at Birmingham, 845 19th Street South, Birmingham, AL 35294-2170 USA; 9 Department of Epidemiology and Biostatistics, UCSF: University of California, San Francisco, San Francisco, CA USA

**Keywords:** HIV, Gender based Violence, Uganda, Intimate partner violence screening, Barriers and facilitators, Theoretical domains framework

## Abstract

**Background:**

People living with HIV are vulnerable to gender-based violence (GBV), which can negatively impact HIV treatment outcomes. National guidelines in Uganda recommend GBV screening alongside HIV treatment services. We explored barriers and facilitators to providers implementing GBV screening and referral in public antiretroviral therapy (ART) clinics in Uganda.

**Methods:**

We conducted qualitative in-depth interviews. Providers were purposively sampled from 12 ART clinics to represent variation in clinical specialty and gender. We used the Theoretical Domains Framework to structure our deductive analysis.

**Results:**

We conducted 30 in-depth interviews with providers implementing GBV screening and/or referral. Respondents had a median age of 36 (IQR: 30, 43) years and had been offering post-GBV care to clients for a median duration of 5 (4, 7) years. 67% of respondents identified as female and 57% were counselors. Facilitators of GBV screening and referral included providers having access to post-GBV standard operating procedures and screening tools, trainings offered by the Ministry of Health, facility-sponsored continuing medical education units and support from colleagues. Respondents indicated that referrals were uncommon, citing the following barriers: negative expectations regarding the quality and quantity of referral services; lack of financial resources to support clients, facilities, and referral partners throughout the referral process; and sociocultural factors that threatened client willingness to pursue post-GBV support services.

**Conclusions:**

Findings from this evaluation support the refinement of GBV screening and referral implementation strategies that leverage facilitators and address barriers to better support individuals living with HIV and who may have heightened vulnerability to GBV.

## Background

The United Nations defines gender-based violence (GBV) as any act of violence directed towards an individual on the basis of their gender, and which results in physical, sexual or psychological harm [1]. While evidence indicates that women are disproportionately affected, men and gender diverse individuals also experience GBV. Intimate partner violence (IPV) is the most common manifestation of GBV and refers to acts or threats of harm that occur between individuals in an intimate relationship [1]. The World Health Organization (WHO) reports that 27% of women aged 15–49 experience lifetime, and 13% of women experience past-year physical or sexual IPV [2]. National estimates from Uganda eclipse global averages—with 39% of Ugandan married women and men indicating that they had experienced physical, sexual, or emotional violence in the past year [3]. Among ever-partnered women, the National Survey on Violence in Uganda estimates that 22% report having experienced physical, 28% sexual and 36% emotional violence in the past year [4]. IPV exposure has been associated with poor mental and physical health outcomes including increased risk of depression, suicidality, HIV acquisition, and poorer engagement with HIV services [5].

In alignment with the Uganda Domestic Violence Act of 2010 and the President’s Emergency Plan for AIDS Relief (PEPFAR) guidance to screen for GBV in HIV treatment and prevention settings, the Uganda Ministry of Health identifies systemic interventions that address GBV as high-priority strategies for reducing the risk of HIV acquisition and supporting retention in care for people living with HIV [6–9]. The Uganda Consolidated Guidelines for the Prevention and Treatment of HIV and AIDS highlight the interconnected nature of GBV and HIV by outlining national recommendations to routinely offer GBV screening and referral to clients in antiretroviral therapy (ART) clinics [6]. Given the co-occurring nature of GBV and HIV, ART clinics represent an opportune environment for identifying and supporting IPV-exposed individuals. Providers play a key role in identifying and supporting ART clinic clients who may be disproportionately vulnerable to GBV exposure because of positive HIV serostatus. Although screening in healthcare settings has been found to increase identification of individuals experiencing violence, only an estimated 10% of providers routinely screen for IPV, globally [10–12]. There is some evidence that provider-level barriers to GBV screening in Ugandan healthcare settings may include GBV stigma; lack of screening knowledge or training; and perceptions that screening is beyond their professional scope [13]. However, factors influencing the implementation of GBV screening and referral guidelines alongside HIV services are under-articulated in Uganda and other resource-constrained settings. Thus, we aimed to identify barriers and facilitators to GBV screening and referral by providers in public ART clinics in Uganda.

## Methods

### Study design

We conducted individual, semi-structured in-depth interviews (IDIs) with providers in 12 ART clinics in the Kampala and Wakiso Districts of Uganda—the two most densely populated districts in Uganda’s Central Region. Participating ART clinics were in urban or peri-urban settings. Further, all health facilities had a designation of Health Centre III or greater (i.e., serve catchment areas of > 20,000 people and are the lowest level at which GBV screening is offered) and were affiliated with the Partners PrEP Program (PPP; #NCT03586128)—the parent study which was an implementation trial that integrated oral pre-exposure prophylaxis (PrEP) and ART delivery [14]. PPP provided the research team with a foundation of rapport with facility administrators and providers as well as understanding of clinic processes.

### Recruitment

Our study team engaged facility leadership and solicited their recommendations of providers suitable for study participation. We then approached these providers to describe the study and gauge interest to participate in a one-time interview. Providers were eligible to participate if they were: engaged in GBV screening and/or referral; proficient in English; willing and able to provide informed consent and 18 years of age or older. We purposively recruited providers and sought representation across the spectrum of clinical specialties, (i.e., counselors, medical officers, nurses, outreach, and linkage facilitators) and gender (i.e., male or female).

### Data collection and analysis

Authors DT (United States-based female) and AN (Uganda-based female) used a semi-structured interview guide to conduct IDIs in English or Luganda, depending on respondent preference. We used the Theoretical Domains Framework (TDF) [15, 16] to guide data collection and analysis. The TDF is an implementation science determinants framework that integrates 33 behavior change theories by characterizing their key factors into 14 domains (e.g., knowledge, skills and social influences) that facilitate the elaboration of cognitive and behavioral influences upon implementation success [15]. We selected this framework because we identified it is as being well-suited for evaluating behavioral influences on providers’ implementation of GBV screening/referral. The research team collaboratively developed the interview guide, which was informed by the TDF, literature reviews, and prior understanding of provider behaviors. Our interview guide explored 11 out of 14 TDF domains (i.e., the optimism, intentions and goals domains were excluded) deemed most relevant for assessing barriers and facilitators to GBV screening/referral (Table 1). IDIs were audio recorded, translated into English (where applicable), and transcribed.

**Table 1:** Theoretical Domains Framework domains [15, 16] and descriptions as applied to the present analysis

TDF domain/code	Domain/code definition
Knowledge	Awareness of information about GBV screening/referral including related concepts, guidelines and best practices related to GBV screening/referral implementation
Skills	Ability or proficiency acquired through practice including the respondent’s perception of their ability to implement GBV screening/referral
Social/Professional role and identity	A coherent set of behaviors and personal qualities related to an individual’s role in GBV screening/referral implementation
Beliefs about capabilities	Respondent’s perceptions about their ability to offer GBV screening/referral to clients
Beliefs about consequences	Expectations regarding what will happen if respondent does (or does not) engage in GBV screening/referral
Reinforcement	Motivating (or demotivating) factors related to engaging in GBV screening/referral
Memory, attention and decision processes	Respondent’s perceptions about factors that guide or influence their decision to offer GBV screening/referral to clients
Environmental context and resources	Any circumstance of a person’s situation or environment that discourages or encourages engagement in GBV screening/referral implementation
Social influences	The interpersonal processes that influence respondent engagement with GBV screening/referral
Emotion	Feelings that respondents have about engaging in GBV screening/referral
Behavioral regulation	Anything aimed at managing or changing objectively measured behaviors related to GBV screening/referral

We used a deductive approach to data organization and analysis which was informed by recommendations for leveraging the TDF for qualitative research [17]. We developed our study codebook by incorporating TDF domains as codes, adapting publicly available TDF domain descriptions to our evaluation context and including illustrative examples for codes. We went line-by-line applying domains of the TDF as codes to transcripts (Table 1). When excerpts were relevant to multiple domains, they were cross coded. The coding team consisted of three US-based female researchers (DT, MR and MM), all of whom possess advanced qualitative research methods training. Four transcripts were jointly coded to refine codebook definitions and promote agreement across the team. Remaining transcripts were independently coded using the finalized codebook. Code application was then audited by another team member. Discrepancies were identified, discussed, and collaboratively reconciled. Upon conclusion of transcript coding and auditing, we generated reports for each TDF domain which summarized data and highlighted key barriers and facilitators for each domain. We conducted two analysis workshops with the broader study team during which we reviewed, discussed and collaboratively built consensus regarding the interpretation of salient study findings. We used Dedoose Software (Version 9.0.86) for data management and analysis [18].

## Results

### Participant characteristics

From August to December 2022, we conducted 30 in-person IDIs with providers in 12 public ART clinics (median interview duration: 56 min, interquartile range (IQR): 45, 68]). Most (67%) respondents were female, median age was 36 years (IQR: 30, 43), and providers had been offering post-GBV care to clients for a median of 5 years (IQR: 4, 7, Table 2). 57% of respondents were counselors, 20% medical officers, 13% outreach or linkage facilitators and the remaining 10% were nurses. 77% of respondents had attained a bachelor’s degree or higher level of education.

**Table 2:** Participant characteristics

Characteristic	N = 30
Time implementing GBV screening/referral, median (IQR) years	5 (4, 7)
**Gender—no. (%)**	
Male	10 (33%)
Female	20 (67%)
Age, median (IQR) years	36 (30, 43)
**Provider type—no. (%)**	
Medical officer	6 (20%)
Nurse	3 (10%)
Counselor	17 (57%)
Outreach	4 (13%)
**Educational level attained—no. (%)**	
Primary	1 (3%)
Secondary	1 (3%)
Tertiary	5 (17%)
Bachelor’s degree	17 (57%)
Postgraduate	6 (20%)

### Theoretical domains

Findings are outlined below and organized by the 11 TDF domains explored in this analysis (e.g., knowledge, skills, social/professional role and identity). In some instances, domains were co-occurring, so relevant findings concurrently presented.

#### Knowledge

Respondents reported a high level of knowledge about GBV. Participants demonstrated dynamic conceptualizations of GBV that were gender-inclusive and included financial control as well as social harms including stigma.

*We are looking for mental. We are looking for physical. We are looking for sexual. Any form of GBV… So you find that it is very hard to find [a client] that is not undergoing GBV. We look out for all that and we ask, “If your family members are stigmatizing you…"”* —Counselor, 34-year-old, Female.

Respondents expressed knowledge about how GBV exposure might influence HIV-related health outcomes.

*We believe that this GBV and HIV, they are like sister and brother. Now, like in Africa… here in Uganda you’ll find that actually GBV has caused HIV… You find that somehow these two are getting related, a lot of GBV has caused HIV. GBV is speeding up the transmission of HIV from one partner to the other.”* —Counselor, 40-year-old, Female.

Participants were aware of screening guidelines and screening tools used to identify and characterize client experiences of GBV. Although participants generally perceived themselves as possessing the necessary knowledge about screening guidelines, they reported some instances in which providers had uncertainty about the appropriate classification of GBV. This may present inconsistencies in GBV categorization across providers and otherwise threaten the utility of GBV screening data.

*For example, the person says, “I’m psychologically tortured because I lost a job…” No one is telling her to be poor or always abusing her, but the person just says, “How will I survive? Where can I get money from?” Where is the GBV in this situation?… Who is the perpetrator? Who is hurting her? There is no one, it is just the situation that is hard for that person. Therefore, that is not GBV… But some people may think that is GBV, because the person is psychologically affected. The person can [classify] it as a GBV, but that’s not a GBV. Especially new staff, when they have just come and they don’t know, sometimes they categorize that as psychological [violence].”* —Counselor, 45-year-old, Female.

Although respondents indicated that they possessed knowledge about GBV screening, some highlighted concerns regarding its implementation in health facilities.

*In screening there are no [knowledge] gaps. Maybe in implementation because not everyone does screening for IPV. It is recommended that at every service point, we do screening for IPV for all clients, but it is not the case. Many go unscreened.”* —Counselor, 49-year-old, Male.

One referral facilitator was having awareness about guidelines, including how and where to refer clients. Providers identified the police as the most common referral partner. Gaps in knowledge, particularly about the existence of referral partners and their service offerings, may pose barriers to referral.

*I know there are many institutions that give different services, but if I’m ignorant about them, then it is like they are not there because the client will not benefit.”* —Counselor, 47-year-old, Male.

#### Skills

Providers reported that they built GBV screening/referral skills in trainings sponsored by the Ministry of Health and through continuing medical education (CME) modules organized by health facilities. CMEs were recognized as important informal training opportunities for GBV screening/referral skills-building. Respondents indicated that: (1) many providers were unable to attend official trainings, and (2) untrained new providers were transferred into facilities and trained providers transferred out. Respondents indicated that CMEs were a facilitator that ensured more providers acquired the skills to support GBV screening/referral implementation. Furthermore, respondents indicated that GBV trainings were largely theoretical and that their skills for GBV screening/referral were largely honed experientially and with support from colleagues.

*I have not attended any training… However, I have learned a lot from my colleagues, and they have taught me a lot of things. Everything I do has been [experiential] job training… My supervisor, who happens to be a clinician, showed me how to identify patients who are experiencing IPV.”* —Counselor, 31-year-old, Female.

Respondents shared perceptions about the importance of rapport building and establishing trust with clients. Participants indicated that these skills facilitated improved understanding of clients’ GBV experiences and better equipped providers to support clients.

*When you create a rapport with them, it encourages them to tell a story. Someone trusts you and they see you as a professional who’s going to help them overcome what they’re going through. Because in the long run, someone feels safe to tell you their story because they know you can significantly change it to a certain extent…. It helps to build the trust and makes the space very safe for them to tell their story without being judged.”* —Counselor, 29-year-old, Female.

#### Social/professional role and identity

All respondents perceived screening clients for GBV as part of their professional role, which represents an important facilitator for screening. Only certain types of providers saw GBV referrals as professionally in-scope. Respondents largely perceived counselors as responsible for connecting clients to referral services.

*It is my superiors [counselors] that refer… to police and other rehabilitation centers. I get cases from the community and hand them over to my superiors.”* —Outreach worker, 49-year-old, Male.

#### Beliefs about capabilities — emotion

The *beliefs about capabilities* and *emotion* domains emerged together. Respondents perceived themselves as capable of enacting GBV screening. They cited trainings, mentorship and prior experiences screening clients as important facilitators for enhancing GBV screening capabilities.

*I feel I am capable. I have dealt with GBV clients for so long. That helps me be a capable person, and then through the trainings, through dealing with GBV on the job, the mentorship… I feel I really know.”* —Counselor, 32-year-old, Female.

Respondents reported difficulty screening male clients, young clients, and clients who had experienced sexual violence. Despite respondents indicating that they felt capable of GBV screening and referral,, respondents cited cultural factors related to gender expectations, stigma, and fear of judgment as barriers to successful screening.

*On the side of the men, we assume that [GBV] goes on and that it is frequent. But the rate at which they disclose is still low. Now, because of that, even our efforts to support them hits a deadlock. I don’t know what causes this, but I want to point a finger at the tradition and the culture. The culture, traditionally, it trains the man to be a hardened man. That even if you have problems, you don’t say them out loud… A real man, even if you are in pain, you don’t cry. Maybe it is from that background… that, “Even if you are a man who undergoes GBV… don’t say it [out loud].“”* —Counselor, 47-year-old, Male.

While participants largely indicated that they were capable of referring clients to post-GBV support services, they identified barriers to referral, including inadequate quality of services offered.

*Of course, on our side it wouldn’t be a challenge, but the client must consent… that it is okay to be given a referral. But sometimes, even if [the client] has consented, the services on the other side are not provided as expected. Those are the challenges. It is challenging to refer. You are referring someone, but in the back of your mind, you are aware that the services are not properly provided [by the referral partner].”* —Counselor, 45-year-old, Female.

When respondents perceived themselves as effectively screening clients for GBV, they reported positive emotions within themselves and within their clients.

*When you screen properly and then when they air out their problems and take your advice, when that client is happy, it also brings happiness to you. Then you feel proud of what you have done. I feel happy when a client’s problem is solved especially when they were thinking that it was impossible to solve it.”* —Medical officer, 41-year-old, Female.

Conversely, respondents indicated that they felt distress if they perceived themselves as being incapable of fully addressing client concerns related to GBV.

*I dread seeing the patients, because it reminds me every time that I’ve actually not been able to do something tangible about it… [the client] is actually going back to the same environment and the same circumstances. I don’t think that’s really changing their situation. Yes. It makes me feel really bad.”* —Medical officer, 27-year-old, Female.

#### Beliefs about consequences — emotion

Respondents underscored the facilitating nature of positive expectations regarding outcomes of GBV screening/referral. For example, participants indicated that they believed screening clients for GBV resulted in clients: (1) gaining awareness about the violence they are facing, and (2) receiving social support to interrupt experiences of violence that might undermine ART adherence and overall health.

*Screening for GBV first makes the patient aware that they’re actually going through something that is not normal. I think that [awareness] is always a key point to start with if we are going to go towards healing and preventing further episodes of violence. It puts the patient in a state of awareness of, “Oh, actually this thing is happening to me, but it’s not normal. But then also the provider has recognized that I’m going through this problem.” They also feel like they’re not alone in their problem. They feel like they have a partner, someone who they can talk to and be helped to overcome that problem.”* —Medical officer, 33-year-old, Female.

Respondents expressed favorable expectations for the ways in which GBV screening/referral influenced providers and facilities. Namely, participants cited that engaging in GBV screening: (1) refined their GBV knowledge and skills via experiential learning, (2) conferred feelings of contentment that they had successfully supported their client, and (3) improved facility responsiveness to key issues of violence affecting clients.

*Yes, of course [screening] helps us as a facility in our decision making and planning purposes to know where we should increase our efforts. If we are having many cases from the catchment area that we are working in, then we see how to intensify the awareness in the area that you are working.”* —Counselor, 32-year-old, Male.

Participants reported negative expectations for patient outcomes if they failed to offer GBV screening. Respondents indicated that failing to screen and support GBV-exposed clients could threaten progress towards addressing the HIV burden.

*We are looking at 2030 as the time to end HIV/AIDS. Therefore, we have that in mind with everything that we do. Now, if there is a practice that deviates us from achieving zero infection by 2030, then we must aggressively work to eradicate it. In a situation where you don’t screen… GBV can happen which can make someone vulnerable to HIV. Therefore, if you don’t give attention to GBV… If you don’t probe, you will not be able to support the client and the client can progress or seroconvert to HIV infections… It is very important. Especially in the fight against HIV, especially new infections.”* —Counselor, 47-year-old, Male.

Respondents reported negative expectations related to offering clients referrals. Participants also reported concerns about offering clients referrals that might not help them address their GBV-related issues. This may represent a barrier to providers offering clients referrals.

*At the end of the day you know, “If I’m sending them to [referral partner], they’re not going to help them. “”* —Counselor, 29-year-old, Female.

The *beliefs about consequences* and *emotion* domains emerged together. In instances in which a referral was believed to result in unfavorable client outcomes, participants indicated that they experienced negative emotions.

*Some people need help and yet where you refer them, they are still not helped. That breaks my heart.”* —Outreach worker, 40-year-old, Female.

#### Reinforcement

Respondents indicated that while there were no official incentives for engaging in GBV screening/referral, an important facilitator was the satisfaction providers received when clients offered feedback indicating that they had been supported. Respondents expressed perceptions that engaging in GBV screening/referral was motivating because it aligned with successful execution of their professional role and contributed to individual as well as community wellness.

*For me it is just the ability to be treated as a worthy person is what makes me screen for gender-based violence… what motivates me is that general wellbeing of a community… I think it’s a very, very pressing thing on my heart that my client gets better. Because if they don’t, it’s going to be bad for all of us… It’ll be bad in terms of adherence. They won’t swallow [HIV medication], so they’ll get high viral load. It’ll be bad in terms of the economy. If they’re sick, they can’t work… It’s the thought that this one individual can actually make the health system or the economy collapse. I think that is my motivation. I really want to see them well, not just medically, but holistically as an individual.”* —Medical officer, 27-year-old, Female.

#### Memory, attention, and decision processes

Most respondents indicated that they did not have to make decisions about whether to screen clients for GBV because all clients were supposed to be screened. Some respondents indicated that overwhelming client volume was a barrier to screening all clients in the ART clinic.

*I miss out on [screening] some because sometimes when clinic is heavy, I find myself not going through everyone, but… I must do it for everyone that comes in. Yes. And then maybe physically you can easily see someone presenting with bruises and then someone has maybe swollen lips and then you want to probe more what is happening with this client.”* —Medical officer, 29-year-old, Male.

Respondents indicated that referrals were, in general, a rare occurrence. Participants noted that referrals were provided via joint decision-making between provider and client and depended upon the nature of the violence as well as client willingness to accept a referral. Respondents identified clients’ unwillingness to accept and pursue referral services as an important barrier.

*We rarely refer. Because most of the things that the clients are facing… Like the perpetrators—those people who mistreat them are their family members, neighbors, or relatives. It is rare… The client will say, “You are going to arrest my husband? But who will provide the food? Who will give school fees to my children? Even if he’s doing such things [using violence], let him be around to work.“ Again, you are referring them, but still they’re not being supported.”* —Medical officer, 45-year-old, Female.

#### Environmental context and resources — behavioral regulation

Some respondents identified understaffing and staff transfers as barriers to offering GBV screening to all clients in ART clinics.

*Most of the health facilities are understaffed so human resource is a factor. If we had enough human resource, it would be easier to screen almost everyone.”* —Counselor, 49-year-old, Male.

Participants identified the following material resources as GBV screening facilitators: screening tools, registry for documenting affected clients, and standard operating procedures outlining guidance for managing clients experiencing violence. Respondents identified the following resources as GBV referral facilitators: GBV referral directory and standard operating procedures outlining referral partners and their contact information. The *environmental context and resources* and *behavioral regulation* domains frequently manifested alongside one another. This is reflected in respondents identifying guidelines and screening tools as facilitating resources for systematically guiding them to implement GBV screening/referral.

*We screen [clients] using the guidelines. Those are guided steps. We have a… GBV screening tool that finds out if you are having any issues.”* —Medical officer, 35-year-old, Female.

*We have a protocol which guides on the referral and linkage. The protocol is very clear, and it is able to guide you on the processes that you take when referring a client.”* —Counselor, 36-year-old, Male.

Some respondents indicated that information in referral directories was static and that the contact information and details of services offered by referral partners were sometimes outdated. This represented a barrier to referral. Participants identified the following as facilitators for referral: (1) phone airtime for contacting referral partners and/or following up with clients, and (2) colleagues to share the responsibility of GBV screening. Respondents identified barriers to referral, including: (1) financial burden to clients of pursuing referrals, (2) lack of financial resources at the facility to ensure successful client referrals, and (3) referral partners lacking the necessary financial resources to adequately support clients.

*Clients normally say the police ask for money and they don’t have the money. Yeah, so they say to win a police case, you really must be loaded. Because those guys will ask for money.”* —Counselor, 34-year-old, Female.

Finally, respondents identified quality referral partners as a limited resource. Participants cited referral partners’ having a narrow scope and mandates regarding the types of clients served as potential barriers to successful referrals.

*Platforms where someone can run for help… There are few and some have limitations. There are those who say they only look after children. Those who say, “No, only women, or this age group.” They leave out other age groups yet every person in this world goes through GBV and they will need to be helped at certain moment.”* —Counselor, 29-year-old, Male.

#### Social influences

Providers identified influences on clients’ willingness to undergo GBV screening and referral including awareness of GBV, fear of being judged because they experienced GBV, shame that community members will learn that a family member caused them harm, gender expectations, and clients requiring consent from a violent partner to pursue additional support services. Respondents indicated that these sociocultural norms undermined client willingness to disclose experiences of violence which posed a barrier to GBV screening/referrals. For example, respondents described how male clients might be judged or emasculated for “coming out” as GBV survivors and participants underscored the barrier that this creates for supporting such clients.

*The males tend to shy away from disclosing certain information… actually there is even a knowledge gap… Usually, males, it takes them longer to disclose or find it hard disclosing. Because we usually feel superior… that ego kind of thing. So they would feel small to admit that they are going through certain kind of GBV.”* —Counselor, 32-year-old, Male.

*The gentlemen, I think, are totally against it. To send them to a safe place or if you send them to police they will not go. Because if they go, [the police] will make them feel emasculated.”* —Medical officer, 29-year-old, Female.

Respondents indicated that collaboration and support from colleagues was an important facilitator to GBV screening and referral. Respondents indicated scenarios in which they might enlist support from a colleague, including: (1) if they had previously experienced GBV and the client’s circumstance was especially resonant or overwhelming, and (2) if they believed the client might have better rapport with another provider.

*You’ll find that, even you, a counselor, as a human being, at one point even you have fallen victim of GBV. For the cases that you feel you cannot handle, or that you feel bring a lot of emotion… If another person says like, “You talk to this person. And I think I may not be able to [manage this case].” Because some cases are similar to even what maybe… you have also fallen a victim to such things.”* —Counselor, 40-year-old, Female.

Respondents indicated the importance of a strong referral network and outlined the ways in which a deficient referral network might pose a barrier to referring clients by instilling distrust in providers and threatening client willingness to accept referral. Some providers indicated that staff turnover within referral agencies compromised referral quality and necessitated reestablishing rapport with a new referral partner.

## Discussion

We aimed to qualitatively assess barriers and facilitators to providers implementing national and multinational guidelines for GBV screening and referral in public ART clinics in two Ugandan districts. Respondents indicated that they routinely implemented GBV screening and referral in ART clinics and that implementation of GBV screening was facilitated by formal and informal training opportunities. Participants cited support from colleagues as a facilitator for GBV screening and referral. Trainings and peer support reinforced theoretical knowledge of best practices to support clients experiencing GBV. These findings are substantiated by existing evidence identifying experiential learning as a facilitator to providers developing the knowledge, confidence, and skills to support clients with a GBV history [19–21]. While providers generally indicated that they were capable of implementing GBV screening, many identified barriers to the provision of GBV referrals, particularly to police. Despite respondents’ perception that GBV referrals were facilitated by their skills, motivation, and a generally enabling resource environment, participants indicated that it was uncommon for them to refer clients.

Our findings are well aligned with those from a 2021 evaluation of the integration of GBV screening into HIV counseling and testing services in Tanzania and South Africa [21]. This evaluation similarly found that despite a high burden of GBV-exposed clients, only 10% of clients experiencing GBV were ultimately referred. Providers from our study indicated that the rarity of referrals was influenced by sociocultural factors and negative expectations about clients being supported by referral partners. Respondents expressed concern that referrals might result in the client sustaining additional institutional harm with little assurance that they would receive meaningful assistance. Providers highlighted concerns about financial constraints associated with pursing referral services and referral agents stigmatizing clients. Our findings are corroborated by existing evidence identifying these barriers as critical contributing factors to the gulf preventing clients with a GBV history from receiving support services [22–24]. When referring clients to post-GBV support services, respondents identified institutional failures that conferred ancillary layers of harm upon already vulnerable clients. This highlights the challenge of providers being capable, willing and appropriately resourced to offer referrals but their efforts being nonetheless compromised due to clients’ (perhaps, justified) resistance or inability to accept support. Further, perceptions of the institutional harm sustained by clients contributed to providers’ negative expectations about referral provision which may have, in turn, posed a barrier to referral by eroding provider willingness to refer and/or exacerbating vicarious trauma.

Respondents identified aspects of the sociocultural context as barriers to GBV screening/referral. Participants highlighted ways in which harmful gender norms operate as tools of systemic sexist oppression by inhibiting client willingness to accept referrals. For example, due to economic reliance upon male partners or family members, respondents indicated that female clients may forego referrals—choosing, instead, to endure environments of ongoing violence out of necessity for their economic survival. This may point to an opportunity for providers to equip female clients experiencing GBV with skills to deescalate violent scenarios. Relatedly, participants theorized that male clients’ willingness to be screened or referred was compromised by fears of being stigmatized for the sociocultural narrative violation of being a male with a personal GBV history. Existing evidence highlights ways in which stigmatizing interactions—including fear of such encounters—may encourage individuals undergoing GBV to refuse social support, thereby reducing their likelihood of engaging in help-seeking behaviors [25–27]. Our findings echo those of Barnett et al. (2016), who identify stigma as a mechanism for social control. Specifically, they indicate that for individuals with a GBV history, individual, interpersonal and systemic participation in stigma processes work to reinforce the existing social order of male dominance [28]. Our observations underscore the importance of ensuring that providers have awareness about the ways in which sociocultural influences may influence clients’ engagement with GBV screening/referral as well as provider ability to support clients given constraints imposed by the broader social environment. Further, these findings point to the importance of mobilizing a community response that extends beyond the health facility and promotes a restructured sociocultural context that allows for more effectively connecting individuals to supportive services for GBV.

Our study findings should be contextualized by considering its limitations. We sampled respondents from ART clinics located in public health facilities. These findings may not be applicable in other geographies or to different types of providers (e.g., at private facilities, lower-level health facilities, other health specialties) or who otherwise possess a less favorable attitude to GBV screening/referral. Further, it is possible that our findings are negatively influenced by social desirability bias. Respondents may have been fearful to share their true experiences of GBV screening/referral due to concerns about being reprimanded for suboptimal performance. Such fears may have compelled some respondents to provide misleading accounts of their GBV screening/referral practices and experiences, although respondents did share numerous challenges. To combat issues of response bias, we assured respondents during informed consent procedures that any information provided would be used solely for research purposes and that no identifying information would be shared with their employer. Another limitation to consider is that we did not capture client-level perspectives about the implementation of GBV screening and referral. Deepening our understanding of client perspectives will be critical for optimizing the effectiveness and impact of GBV screening and referral in ART clinics.

## Conclusions

There is limited description of factors influencing the implementation of GBV screening and referral guidelines that have been enacted in Uganda with the goal of organizing a health systems response to concurrently address challenges of GBV and HIV. In this qualitative evaluation among providers in ART clinic settings, we identified several barriers and facilitators to offering GBV screening and referral. Given the disproportionate burden of GBV among individuals living with HIV, it is important to implement bespoke strategies for addressing issues of violence within the context of HIV care. It will be advantageous to develop implementation strategies that enable providers to navigate threats that the sociocultural environment poses to GBV screening and referral and address resourcing challenges that inhibit referral network effectiveness. Future research is needed to incorporate client-level perspectives and evaluate dynamic strategies for enhancing the health systems response to GBV by addressing issues of violence in community settings.

## Data Availability

Not applicable. In this qualitative evaluation, we ensured participants that their data would remain confidential and would only be shared with our study team. Thus, we will not make interview transcripts available to external parties.
